# The Mediation of Care and Overprotection between Parent-Adolescent Conflicts and Adolescents’ Psychological Difficulties during the COVID-19 Pandemic: Which Role for Fathers?

**DOI:** 10.3390/ijerph20031957

**Published:** 2023-01-20

**Authors:** Barbara Forresi, Ludovica Giani, Simona Scaini, Giampaolo Nicolais, Marcella Caputi

**Affiliations:** 1 Department of Psychology, Sigmund Freud University (Milan), Ripa di Porta Ticinese, 77-20143 Milan, Italy; 2 Department of Dynamic, Clinical and Health Psychology, Sapienza University of Rome, Via degli Apuli, 1-00185 Rome, Italy; 3 Department of Life Sciences, University of Trieste, Via E. Weiss, 2-34128 Trieste, Italy

**Keywords:** adolescent, parent-adolescent conflict, care, overprotection, parenting, mental health, emotional difficulties

## Abstract

Background: There is evidence of a significant raise in youths’ emotional and behavioral difficulties during the pandemic. Only a few studies have addressed parent-adolescent conflict, and none investigated the possible mediating effect of parenting in the association between conflicts with parents and adolescents’ symptoms. This study aimed at investigating youths’ psychological symptoms during the pandemic, focusing on the predicting effect of parent-adolescent conflict. The mediating role of care and overprotection was also explored, considering whether adolescent gender moderated this mediation. Methods: 195 adolescents aged 14–18 years participated in an online longitudinal study. Perceived conflict with parents and parenting dimensions (Parental Bonding Instrument; PBI) were assessed at baseline (2021). Self-reported psychological difficulties (Strengths and Difficulties Questionnaire; SDQ) were collected at baseline and after one year (2022). Results: A significantly severer symptomatology was found in adolescents having a conflictual relationship with one or both parents. Major conflicts with parents correlated with lower care and greater overprotection in mothers and fathers. However, parental overprotection and maternal care were not mediators of the relationship between conflict and youths' difficulties. The only exception was represented by paternal care that fully mediated this relationship in both adolescent males and females. Conclusions: Although further investigations are needed to overcome limitations due to the small sample, findings extend our insight into the impact of parent-adolescent conflict, highlighting the role of fathers’ care and the need to maximize their involvement in clinical interventions.

## 1. Introduction

Even before the COVID-19 pandemic, a significant burden of mental health disorders on adolescents was widely reported, with psychological difficulties affecting 10 to 20% of youths in the world [1,2], and epidemiological evidence supporting a higher prevalence of emotional problems [3,4].

Increasing trends were observed during and after the pandemic in longitudinal studies [5,6], reviews and meta-analyses [7,8,9], also in Italian samples [10,11,12,13]. Measures adopted to contain the spread of the virus, like stay-at-home orders, lockdowns and quarantines, social distancing, and online schooling, along with fear of illness, put teenagers’ psychological well-being at risk, contributing to a raise in anxiety and depression. Not only adolescents suffered from a lack of social and peer support, but the time spent at home, for many of them, was also associated with heightened family conflicts, stress, and irritability, especially due to financial challenges and job losses [14,15,16].

Several studies have shown that parent-child conflicts during the pandemic period were associated with poor mental health in youths [16,17,18,19,20,21,22,23,24,25], confirming the role of proximal factors in shaping youths’ adjustment. Chan [22] observed that the relationship between COVID-19 stress and lower levels of well-being, both in parents and children, was mediated by parent-child conflict. The parent-child conflict was acknowledged as the strongest predictor of child-perceived stress/negativity [23], adolescents’ substance use [26], and a significant risk factor for adolescent adjustment in longitudinal studies [16,21]. Evidence collected during the pandemic substantiated previous meta-analyses (e.g., [27]), cross-sectional [28,29,30] and longitudinal studies [29,31,32,33], showing the association between parent-adolescent conflict and adolescents’ psychopathology. However, only a few addressed the father–adolescent conflict [34,35].

The scientific literature also pointed out the key role played by parenting dimensions in youths’ psychological adjustment, with dysfunctional parenting, such as low warmth and high control, more frequently observed in adolescents with psychopathology [36,37]. More specifically, adolescent depression was found to be negatively correlated with parental warmth [38] and positively correlated with maternal psychological control [39]; similarly, adolescent irritability was negatively associated with parental warmth and positively associated with parental overprotection [40]. Other studies highlighted the association between high levels of overparenting and poor psychological well-being among adolescents [41], with higher levels of anxiety and depression [42]. Eun and colleagues [43] observed that maternal high care and low control had a protective role for depressive, eating, and behavioral disorders in offspring; on the contrary, paternal high care and low control protected adolescents from social phobia and dependence. In the same study, gender differences were also acknowledged: maternal overcontrol correlated with anxiety and substance abuse in daughters, whereas paternal overcontrol was associated with substance use in sons. Similar findings were found in a clinical sample of Italian adolescents [44], with parental low care and high control associated with higher levels of psychopathology, especially in girls.

While the influence of both parent-adolescent conflict and parenting care/control on adolescents’ adjustment has been recognized [24,31], much of the research explored these variables in isolation from one another. Specifically, there is scant research on how conflictual parent-adolescent relationships and parenting might interact in predicting developmental outcomes for youths. Although some studies found empirical evidence of a *control-driven model* wherein parental control triggered parent-adolescent conflict, to date, the directionality of the effect at the within-family level is not clear. Leung et al. [45], for example, hypothesized that adolescents might respond to mothers’ overparenting by acting against them, therefore raising levels of conflict. Instead, Sun et al. [46] supported a *conflict-driven model*, according to which when parent-youth conflict raises, an increase in parental overcontrol can be observed as a result (not the reverse). Accordingly, other studies [27,47] showed that higher parent-adolescent hostility could increase intrusiveness and control, reducing positive parenting practices in both mothers and fathers. It was also observed that parent-adolescent conflict predicted parental support: when the conflict was higher, in fact, the support was lower [48]. Other studies focused on parental warmth: while Eisenberg et al. [49] found that the quality of parenting could influence parent-adolescent conflict, Silva et al. [50] observed that parental warmth had a moderating role in the daily association between parent-adolescent conflict and adolescents’ well-being. To our knowledge, no prior studies investigated parental care and control as possible mediators of the relationship between parent-teen conflict and adolescent psychological difficulties.

Disentangling the different components of parent-adolescent relationship quality (conflict) and parenting style (care and control), and further analyzing the relationship between these variables in influencing adolescents’ adjustment, however, could be of great interest. Several researchers [51,52,53,54] observed that restrictions and family changes during the pandemic had a different influence on families, having the potential to bring family members closer and enhance parent-child bonding. On one side, the “stay at home” restrictions and working from home brought additional stress to families, resulting in increased patterns of conflicts; on the other, they predicted an increase in parental warmth, closeness and care, with higher levels of positive affect in youths [16]. Not all conflicts were associated with a negative outcome: different adolescent life change profiles were seen during the COVID-19 pandemic (improved, unchanged, and worsened), and parent-teen conflict was a significant predictor for all these patterns [55].

Therefore, the main objectives of the present study were:

- To investigate youths’ emotional and behavioral symptoms during the pandemic, focusing on the predicting effect of parent-adolescent conflict. With regard to this, it was hypothesized that higher levels of perceived conflicts with parents could predict higher levels of symptoms reported by youths one year later;
- To investigate the association between parenting dimensions (care and overprotection) and youths’ adjustment. It was hypothesized that high parental overprotection and low care would be associated with higher symptoms in youths;
- To explore the mediating effects of parenting dimensions (mother and father care and overprotection) in the relationship between parent-adolescent conflict and youth symptoms after one year. As no previous studies explored this mediation, we did not make any specific hypothesis;
- To explore whether this mediation was moderated by adolescent gender. Even in this case, specific hypotheses could not be pronounced.

## 2. Materials and Methods

### 2.1. Participants

A convenience sample of 195 adolescents aged between 14 and 18 years (M = 16.31, SD = 1.22, 78% females) voluntarily took part in a web-based longitudinal study (T0 = May 2021, T1 = December 2021, and T2 = June 2022). 36% of participants came from Northern Italy, 32.1% from Southern Italy, and 31.9% from Central Italy.

### 2.2. Procedure

The current study focused on data collected at the first (T0) and the third wave (T2) of the broader longitudinal study. At T0 (April 2021), adolescents reported sociodemographic information, perceived conflict with parents and parental behaviors (care and overprotection), as well as their emotional and behavioral symptoms. Psychological symptoms were re-assessed at T2 (May 2022).

While the first assessment took place between April 22nd and May 21st, 2021, in the third wave the questionnaire remained available online from May 22nd until June 21st, 2022. Participants were recruited through a snowball sampling strategy on social networks and were asked to complete an online battery of questionnaires that were developed on Google Forms. Those who adhered to the study and responded at T0 were recontacted at T2 by using the email address voluntarily provided for the follow-up. The third wave of data collection (T2) took place by sending via email the link to the Google Forms questionnaire. Starting from a sample of 1195 subjects at T0, the T2 data collection encountered a significant dropout rate with only 195 adolescents completing the assessment.

The present study was approved by the Ethical Committee of Sigmund Freud University (n. PBZGDX3OAYFCW288612) and followed the ethical standards recognized by the Declaration of Helsinki. All participants received complete information about the study and provided their consent.

### 2.3. Measures

#### 2.3.1. Parent-Adolescent Conflict

Three ad hoc questions were developed to assess parent-adolescent conflict. Participants were first asked whether they had noticed an increase in conflict with parents during the pandemic. Afterward, they were asked if this conflict was with one (mother or father) or both parents. The sum of the conflicts experienced by each participant (none, with one or both parents) provided the total score of conflict (range 0–2).

#### 2.3.2. Parenting

Parental Bonding Instrument-PBI [56] is a widely used 25-item self-report questionnaire to assess parental style and behaviors as perceived by adolescents. This measure was developed by Parker et al. [56], whose theory on optimal and dysfunctional bondings was coherent with the attachment theory [57], as well as with Ainsworth et al.’s [58] and Main’s [59] theory. According to Parker and colleagues [56], parental care (i.e., the degree to which the parent is perceived as expressing affection, emotional warmth, empathy, and closeness or, on the other pole, coldness, indifference, and neglect) and parental overprotection (i.e., the degree to which a parent is perceived as controlling, overprotective, intrusive, searching for excessive contact, infantilizing and preventing autonomy or, on the other pole, promoting independence and autonomy) are the two main dimensions of perceived styles of parenting. In the same form, adolescents provide information on mother and father parenting. Its psychometric properties were investigated in previous studies showing good retest reliability, high internal consistency, validity, and stability [56,60,61,62]. The Italian version of the PBI [63,64] showed high internal consistency, with 0.75 for the mother’s care, 0.84 for the mother’s overprotection, 0.83 for the father’s care, and 0.88 for the father’s overprotection. A two-factor model was identified that accounted respectively for 44.6% and 44.3% of the variance of mother and father PBI scores in a sample of students [63].

#### 2.3.3. Emotional and Behavioral Difficulties

The self-report version of the Strengths and Difficulties Questionnaire-SDQ [65], Italian version [66], was used to measure participants’ psychological health. This questionnaire is for young people aged 11-17 years and it is composed of 25 items distributed in 5 subscales of 5 items each: emotional symptoms, conduct problems, hyperactivity/inattention, peer relationship problems, and prosocial behavior. Items are rated using a 3-point Likert scale (not true, somewhat true, certainly true, respectively). The scores of the different subscales (except for the prosocial scale) are summed to generate the total difficulties score, which ranges between 0 and 40 and can be categorized as normal (0–15), borderline (16–19) or abnormal (20–40). Normative data for the Italian population are available: Cronbach’s α for the total difficulties scale of the self-report Italian version of SDQ was 0.52 in the 25-item version and 0.60 in the version without reverse items [66].

### 2.4. Statistical Analyses

Statistical analyses were performed using SPSS software version 26 (IBM Corp, Armonk, NY, USA, 2019). Firstly, explorative analyses were conducted to test whether continuous variables (SDQ and PBI) were normally distributed. Frequencies, percentages, means, and standard deviations were used for descriptive analyses. Secondly, *t*-tests were used to examine gender differences for continuous variables (SDQ and PBI). Pearson’s correlation analyses were conducted to explore the relationships among age, parent-adolescent conflict index, parental overprotection and care, and SDQ scores. One-way ANOVA post hoc Bonferroni test was performed to compare SDQ scores in adolescents with different conflictual levels. PROCESS macro model 4 [67] was used to calculate the mediation effect, with 5.000 bootstrap estimates for the construction of 95% bias-corrected confidence intervals. For all statistical tests, a *p* < 0.05 was considered significant.

## 3. Results

### 3.1. Descriptive Statistics

Among the 195 adolescents, 97 (m = 22, f = 75) reported they did not experience conflict with parents, 50 (m = 9, f = 41) lived a conflictual relationship with one parent, 42 (m = 9, f = 33) with both parents, and 6 did not answer this question (all females). Chi-square analysis outlined the absence of a significant difference between males and females in the three conditions (χ^2^(2) = 0.435, *p* = 0.804).

While 47.4% of the sample had a total SDQ score within the normal range, 18.5% were in the borderline range, and 34.1% reported clinically significant symptoms. More specifically, 16.3% presented borderline behavioral symptoms and 16.3% was in the abnormal range. A different trend emerged for emotional symptoms as the majority of the participants (44.4%) experienced abnormal levels, and 13.3% fell into the borderline range.

### 3.2. Preliminary Analyses

As the first analyses, Shapiro–Wilk tests were performed to assess the normal distribution of the SDQ total score (W(179) = 0.984, *p* = 0.043), PBI maternal care score (W(179) = 0.945, *p* ˂ 0.001), PBI paternal care score (W(179) = 0.965, *p* ˂ 0.001), PBI maternal overprotection score (W(179) = 0.955, *p* ˂ 0.001), and PBI paternal overprotection score (W(179) = 0.954, *p* ˂ 0.001). Furthermore, attrition analyses were carried out to exclude significant differences between the initial and the remaining sample at T2 (after 1 year), including age, gender, and exposure to COVID-19 stressful events. Findings revealed that no relevant differences emerged between the two groups with regard to age (T(1193) = 0.874, *p* = 0.382), gender (χ^2^(1) = 1.788, *p* = 0.181), and exposure to stressful pandemic-related events (χ^2^(1) = 0.001, *p* = 0.974).

Before performing moderated mediation analyses, a series of *t*-tests and correlations were run. In particular, the independent-sample *t*-test revealed significant gender differences, with higher emotional and behavioral symptoms in females (t(168) = −4.406, *p* ˂ 0.001). Furthermore, at the two time points of the study, the one-way ANOVA post hoc Bonferroni test showed that adolescents reporting conflictual relationships with both parents had statistically significantly higher SDQ scores than youths living in non-conflictual families (F = 10.11, *p* ˂ 0.001 at T0; F = 4.53, *p* = 0.013 at T2) Although SDQ scores were higher when conflicts involved both mothers and fathers, no significant differences were found between the conditions “conflict with one” and “with both parents”.

Finally, Pearson correlations were conducted to examine the relationship between age, parent-adolescent conflict, maternal and paternal care, maternal and paternal overprotection, and total adolescent emotional/behavioral difficulties. Table 1 shows Pearson’s correlation results. Significant negative correlations were found between parent-adolescent conflict and parental care, while positive correlations were observed with parental overprotection. The results also showed significant positive correlations between maternal and paternal care and a negative correlation between maternal care and maternal/paternal overprotection. Notably, youths’ symptoms had a strong positive correlation with parent-adolescent conflict and a strong negative correlation with fathers’ care.

### 3.3. Main Analyses: Moderated Mediation Models

To test the effects of T0 parent-adolescent conflict on T2 adolescent emotional and behavioral problems, mediated by T0 parental overprotection/parental care, we assumed a moderated mediation model, described by Hayes [67] as model 14 of mediation, including gender as moderating factor. 

We tested four separate models with four different mediators. In the first model, adolescent-parent conflict was the independent variable (X), adolescent emotional and behavioral difficulties, represented by the SDQ total score, were the dependent variable (Y), maternal overprotection represented the mediator (M), and adolescent gender embodied the moderator (W) on the path between M and Y. In the second model, we tested the same moderated mediation using paternal overprotection as M, whereas, in the third and fourth models, we examined the mediating role of maternal and paternal care (respectively) on the relationship between parent-adolescent conflict and adolescent psychological difficulties, differentiating for adolescent gender.

In the first model, the direct effect of parent-adolescent conflict on maternal overprotection (ß = 2.81, SE = 0.59, *p* ˂ 0.001, 95%, CI [1.65, 3.96]), the direct effect of conflict (ß = 2.23, SE = 0.56, *p* ˂ 0.001, 95%, CI [1.12, 3.34]) and adolescent gender (ß = −4.79, SE = 2.36, *p* = 0.04, 95%, CI [−9.44, −0.13]) on adolescent emotional and behavioral total difficulties were significant. Conversely, the direct effect of the mediator on the outcome (ß = −0.02, SE = 0.07, *p* = 0.81, 95%, CI [−0.16, 0.13]), as well as the interaction effect of the mediator with the moderator on the outcome (ß = 0.15, SE = 0.18, *p* = 0.39, 95%, CI [−0.19, 0.51]), were not significant. A simple slope test revealed that the indirect effect of parent-adolescent conflict on adolescent difficulties through the mediation of maternal overprotection was not significant for either females (ß = −0.05, SE = 0.23, 95%, CI [−0.48, 0.45]) or males (ß = 0.39, SE = 0.43, 95%, CI [−0.47, 1.25]). As this first moderated mediation model was not significant, the figure was not included.

In the second model, all the direct paths were significant (effect of parent-adolescent conflict on paternal overprotection = 3.21, SE = 0.56, *p* ˂ 0.001, 95%, CI [2.11, 4.32], the effect of paternal overprotection on adolescent emotional and behavioral problems = 0.16, SE = 0.08, *p* = 0.04, 95%, CI [0.004, 0.31], and effect of parent-adolescent conflict on adolescent emotional and behavioral problems = 1.61, SE = 0.59, *p* = 0.006, 95%, CI [0.45, 2.77]). The simple slope test revealed that paternal overprotection did not have a significant indirect effect on total emotional and behavioral difficulties of the adolescent for either boys (ß = 0.43, SE = 0.56, 95%, CI [−0.67, 1.61]) or girls (ß = 0.51, SE = 0.29, 95%, CI [−0.04, 1.11]). Therefore, the first two moderated mediation models outlined that maternal and paternal overprotection are not mediators of the relationship between parent-adolescent conflict and adolescent psychological problems, even though they both directly influence their well-being. As this second moderated mediation model was not significant, the figure was not included.

**Figure 1:**
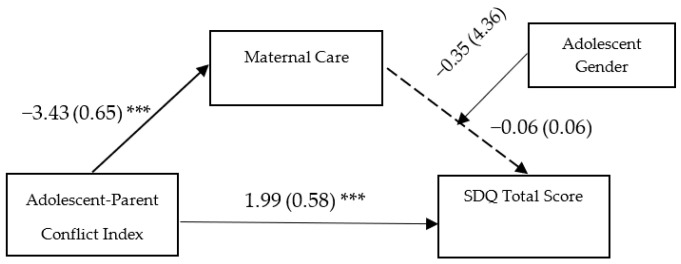
Maternal care as a mediator of the association between Adolescent-Parent Conflict Index and Adolescent Emotional and Behavioral Problems. Unstandardized coefficients are reported with standard errors in parentheses. The model includes Gender as a moderator on the path between maternal care and Adolescent SDQ Total problems. Analyses were based on 5000 bootstrap samples with 95% bias-corrected confidence intervals; *** *p* < 0.001; SDQ = Strengths and Difficulties Questionnaire.

In the third model (see Figure 1), the direct effect of adolescent-parent conflict on maternal care (ß = −3.43, SE = 0.65, *p* ˂ 0.001, 95%, CI [−4.71, −2.15]) and the direct effect of parent-adolescent conflict (ß = 1.99, SE = 0.58, *p* ˂ 0.001, 95%, CI [0.86, 3.14]) on total adolescent difficulties were significant. Conversely, the direct effect of maternal care (ß = −0.06, SE = 0.07, *p* = 0.375, 95%, CI [−0.19, 0.07]) and adolescent gender (ß = −0.35, SE = 4.36, *p* = 0.93, 95%, CI [−8.97, 8.26]) on the outcome, as well as the interaction effect of maternal care with the adolescent gender on adolescent emotional and behavioral total problems (ß = −0.09, SE = 0.17, *p* = 0.57, 95%, CI [−0.42, 0.23]) were not significant. Similar to the previous models, the indirect effect of parent-adolescent conflict on adolescent difficulties mediated by maternal care was not significant in either males (ß = 0.53, SE = 0.52, 95%, CI [−0.23, 1.81]) or females (ß = 0.19, SE = 0.25, 95%, CI [−0.26, 0.74]).

**Figure 2:**
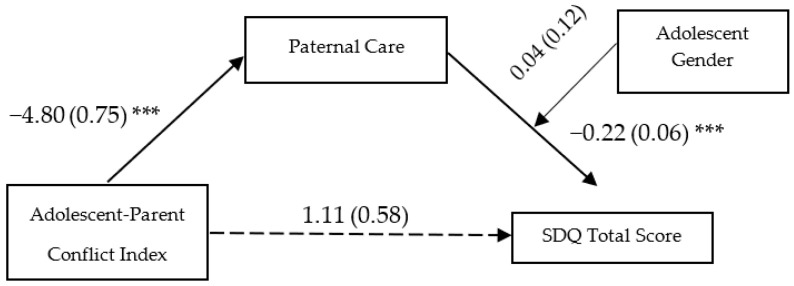
Paternal care as a mediator of the association between Adolescent-Parent Conflict Index and Adolescent Emotional and Behavioral Problems. Unstandardized coefficients are reported with standard errors in parentheses. The model includes Gender as a moderator on the path between maternal care and adolescent SDQ Total problems. Analyses were based on 5000 bootstrap samples with 95% bias-corrected confidence intervals; *** *p* < 0.001; SDQ = Strengths and Difficulties Questionnaire.

In the fourth and last moderated mediation model (see Figure 2), the effect of parent-adolescent conflict on paternal care (ß = −4.80, SE = 0.75, *p* ˂ 0.001, 95%, CI [−6.29, −3.32]) and the effect of paternal care on adolescent psychological difficulties (ß = −0.22, SE = 0.06, *p* ˂ 0.001, 95%, CI [−0.33, −0.114]) were significant. Conversely, the direct effect of parent-adolescent conflict on the adolescent psychological outcome (ß = 1.11, SE = 0.58, *p* = 0.06, 95%, CI [−0.04, 2.26]) was not. The adolescent gender, alone (ß = −3.57, SE = 2.69, *p* = 0.19, 95%, CI [−8.88, 1.74]) and intertwined with paternal care (ß = 0.04, SE = 0.12, *p* = 0.71, 95%, CI [−0.19, 0.28]), did not have a significant effect on the adolescent SDQ total score. Therefore, paternal care fully mediated the relationship between parent-adolescent conflict and the total adolescent score of emotional and behavioral problems for both males (indirect effect: ß = 1.06, SE = 0.34, 95%, CI [0.46, 1.79]) and females (indirect effect: ß = 0.84, SE = 0.47, 95%, CI [0.01, 1.89]). In other words, a high conflict between youths and their parents reduces the ability of fathers to take care of their adolescent children, which, in turn, heightens the likelihood of the adolescent developing emotional and behavioral difficulties.

## 4. Discussion

The present study adopted a longitudinal design to explore the predicting effect of parent-adolescent conflict on youths’ psychological symptoms during the pandemic, and the possible mediating effect of parental care and overprotection, bridging a gap in the literature. To our knowledge, no previous studies have examined this mediation relationship.

Results showed high levels of psychological difficulties in youths, which is consistent with other studies carried out worldwide [5,6,7,8,9] and in Italy [10,11,12,13] during the COVID-19 pandemic. Furthermore, higher symptoms at the SDQ were found in adolescents who perceived a conflictual relationship with one or both of their parents, compared to the non-conflict condition.

The first hypothesis of the study was therefore confirmed: findings supported the important role of conflicts with parents in explaining adolescent adjustment during the pandemic [16,17,18,21,22,23,24,26,28,29]. As observed by Wang et al. [20], the pandemic brought additional stress and increased patterns of conflicts to some families, which in turn predicted higher levels of adolescents’ psychological difficulties. Spending more time together (often in small houses), changes in daily routines (home-working and online schooling), personal losses, job losses and financial crises experienced by families between 2020 and 2021 could have raised levels of family conflicts, including those between parents and adolescents. Findings also confirmed that parent-adolescent conflict might have an influence on youth psychological symptoms over time (a year later), in line with previous longitudinal studies [16,21] performed during the pandemic.

Regarding our second hypothesis, in accordance with prior studies [41,43,44], youths’ symptoms were significantly negatively correlated with parental care and positively correlated with paternal overprotection. However, differently from previous research [39,40], no significant associations were observed with maternal overprotection.

The present study extended previous research on parent-adolescent conflict by conducting moderated mediation analyses (in line with the third and fourth research objectives), whose main finding was that maternal overprotection and care, and paternal overprotection did not mediate the relationship between parent-adolescent conflict and adolescent psychological difficulties. In the first three mediation models, in fact, a direct impact of the conflict on adolescents’ well-being was found that was independent of parenting dimensions.

Unexpectedly and interestingly, the only exception was represented by paternal care (fourth model) that fully mediated the relationship between parent-adolescent conflict and adolescent emotional/behavioral symptoms in both genders. In fact, the direct effect between conflicts with parents and adolescents’ psychological difficulties disappeared when the father’s care was added to the statistical model. Moreover, this observed mediation was not moderated by gender, and no difference was observed between adolescent males and females.

First of all, these results suggested that conflicts with parents can not only affect adolescents’ emotional and behavioral difficulties (both directly and indirectly), but also directly affect parenting, with growing overprotection and lower levels of care, in both mothers and fathers. These findings appeared in accordance with studies supporting a *conflict-driven model* and pointing out that when parent-youth conflict rises, an increase in parental control can be observed [46] while support lessens [48].

Second, the importance of fathers’ care was emphasized: not only paternal care and overprotection had a significant influence on adolescents’ psychological symptoms (in the mediation analyses, the same effect was not observed for mothers), but the negative impact of parent-adolescent conflict on adolescent’s psychological difficulties was fully mediated by lower levels of fathers’ care. The present study seems to suggest that when fathers show less affection, emotional warmth, empathy, and closeness (or more coldness, indifference, and neglect according to Parker’s theory, 56), adolescents—both males and females—do not suffer from the conflict itself but from being deprived of father’s care. 

Several insights might help interpret these findings. Although many studies recognized the importance of fathers’ contribution to parenting [68,69,70,71], there is considerable evidence showing that, compared to mothers, fathers can be less concerned with parenting questions [72] and spend less time with their children [73]; they can be detached from the emotional lives of their sons and daughters [74], less intimate [75], and conflict avoidant [76]. It could be therefore hypothesized that parent-adolescent conflict might further limit their caretaking role and their emotional involvement, having a strong impact on their adolescent children. In the stressful situation of the pandemic, when conflicts could not be avoided (e.g., because of working from home), fathers living conflictual relationships with their offspring might have been significantly affected in their caretaking role, becoming less warm and sensitive, less empathic, or more distant. A previous study, for example, found that fathers might use dysfunctional conflict management strategies, such as withdrawal [77]. Moreover, according to a family system theory [78] and spillover models [79,80], it could be hypothesized that not only fathers-adolescent conflict, but also mother-adolescent conflict (or interparental conflicts) could transfer their effect on fathers’ parenting, having a negative impact on youths’ well-being as a result.

These findings are consistent with earlier studies suggesting the distinctive and significant role of fathers during adolescence [43,68,81,82,83,84]. While their role has long been overlooked in the developmental psychopathology literature, in recent years, research on fatherhood has become an area of increasing interest, with cross-sectional and longitudinal studies exploring their contributions from infancy to adolescence [68]. It was found, for example, that conflicts with fathers, compared to mothers, had a stronger association with youths’ psychological symptoms [6,82] and that attachment to fathers (not to mothers) significantly predicted peritraumatic distress in adolescents during COVID-19 [83].

While this study provides a first contribution to understanding the mechanisms underlying the associations among parent-adolescent conflict, parenting dimensions and adolescents’ psychological difficulties, more research is needed to further explore these findings.

### Strengths and Limits

To the best of our knowledge, this is the first study to explore parenting dimensions longitudinally as possible mediators of the relationship between parent-adolescent conflicts and adolescents’ symptomatology. In line with previous research [e.g., 48], it represented a first tentative to untangle parenting domains (care and overcontrol, according to Parker et al., [56]) and the quality of parent-adolescent relationships (conflict) to better understand how these processes “function in concert with each other, as a prelude to developmental risk for adolescent problem behavior” [48] (p. 1753). Findings provided insights into the relationships among parent-adolescent conflicts, parenting and adolescents’ adjustment, suggesting the important role of fathers’ care in adolescence, especially during stressful times.

Several limits, however, must be recognized. A convenience small sample, the use of online surveys, and the significant dropout rates at the third wave considerably restricted the generalization of the results. Although the online recruitment had the potential to reach a population that might be usually difficult to access, the limited sample size reduced the statistical power of the analyses as well as the possibility to robustly support our conclusions.

The conflictual relationship with parents was assessed only through dichotomous yes/no questions, and an index was developed based on these items. The aggregation of mother-adolescent and father–adolescent conflict into one construct (conflict “with one” or “both parents”) obscured the unique effects of these different relationships. Among the others, frequency, intensity, duration, and conflict resolution strategies were not investigated, limiting to a great extent the comprehension of conflicts. Future studies should better examine these variables, as well as conflict trajectories [31,32,33]. Additionally, no information was collected on parenting status (i.e., married, divorced, living together or in different houses), SES, or about the number of children that might have influenced the results.

Furthermore, no information was collected from parents. Although assessing parenting practices from boys and girls was coherent with studies pointing out that adolescents’ perspectives have a greater association with psychological outcomes than parents’ self-reports [85], youths’ perceptions could be biased. Having parent reports would have been beneficial to gain additional insights: it was found, for example, that discrepancies in parent and adolescent reporting of conflict severity, with lower levels reported by parents, could be associated with higher hopelessness in adolescents [86].

Due to the small sample size, a more detailed examination of parent-adolescent conflict and adolescents’ emotional and behavioral difficulties was not allowed. Further work is needed on father-adolescent and mother-adolescent conflicts (as well as on conflicts in same-sex parent-adolescent dyads), as well as on their association with internalizing and externalizing symptoms in youths.

Lastly, the overrepresentation of females in our sample seems to suggest a major proneness of girls to adhere to studies concerning their well-being and daily difficulties. This gender unbalance suggests that more research is needed to understand male functioning under stressful periods.

Building on this explorative study, future research should continue to investigate the association between parent-adolescent conflict and adolescents’ psychological difficulties by examining mediators and contextual moderators of this association. Parent-adolescent conflict is not necessarily detrimental and could also be associated with improved patterns of development [55]: while a rise in conflict is part of typical family relationships in adolescence [87], under which conditions these conflicts are associated with negative outcomes for parenting, for parent-adolescent relationships, and adolescents’ development is still a matter of debate. Previous studies [27,48,49,88,89,90] significantly contributed to the literature by investigating the role of adolescent characteristics and parenting during conflicts: more research is needed on how parenting dimensions could change or be affected in conflictual relationships, influencing adolescents’ well-being. Additional attention should be provided to fluctuations in parenting behavior and functioning during conflictual interactions, also through dynamic methods and daily diaries [91,92].

Parental psychopathological symptoms should also be included in future investigations [93] as they may impact parenting practices, resulting in less warmth, less sensitivity, and higher hostility [94]. Moreover, it should be considered that while conflicts with parents might lead to lower adolescents’ psychological adjustment, emotional and behavioral problems in adolescents might trigger more conflict with parents [45]. A better examination of this bidirectional impact between conflict and psychological symptoms [95] is therefore needed.

Finally, the interchange between parent-adolescent conflict and interparental conflict should be further investigated [96,97,98,99]: according to studies supporting the spillover hypothesis [79,80], in fact, fathers are more likely than mothers to transfer their negative emotions due to marital conflict to the parent-child system.

## 5. Conclusions

The present longitudinal study showed that parent-adolescent conflict in the pandemic period, also mediated by lower levels of paternal care, adversely affected adolescents’ emotional well-being.

Withstanding limitations, findings provide suggestions that could inform intervention with adolescents and their families. First, programs to foster well-being in youths exposed to stressful situations should consider family-oriented programs with the goal of decreasing conflict and minimizing its negative impact. Conflictual relationships, in fact, may negatively affect communication with parents, which is particularly important when adolescents are dealing with adverse events and rely primarily on family support, being limited their opportunities to socialize with friends (e.g., during the pandemic).

Second, clinicians should maximize their effort in including fathers in interventions. According to the literature [72,73,74,75,76,77], fathers who are not skilled to effectively manage conflicts could reduce their level of care and become less sensitive to adolescents’ developmental needs (e.g., independence and autonomy). Accordingly, they should be helped to use constructive conflict management strategies (e.g., to avoid withdrawal [77]), to keep their focus on adolescent needs, and provide their care and support.

Given the positive association between reflective functioning and parenting [100,101], interventions to enhance mentalizing skills during conflictual parent-adolescent interactions could be particularly effective and suitable. They might be especially important for fathers [101]: an increased reflective functioning, in fact, might foster their awareness of factors that limit the emotional involvement with their adolescent children and could help the whole family cope with negative emotions and conflicts.

Future studies, further exploring the relationships among parent-adolescent conflict, parenting dimensions and adolescents’ mental health, may help elucidate the role of fathers as well as how to promote systemic change within families.

## Figures and Tables

**Table 1:** Correlations Among the Study Variables.

	Mean (SD)	2	3	4	5	6	7
1. Care (M)	24.65 (7.61)	0.512 ***	−0.410 ***	−0.246 ***	−0.189 **	−0.364 ***	−0.108
2. Care (P)	20.08 (9.09)	-	−0.295 ***	−0.424 ***	−0.370 ***	−0.436 ***	−0.082
3. Over protection (M)	12.62 (6.88)		-	0.673 ***	0.095	0.332 ***	0.014
4. Over protection (P)	11.32 (6.71)			-	0.225 **	0.397 ***	−0.048
5. SDQ Total	16.77 (6.24)				-	0.291 ***	−0.033
6. C-P Conflict Index	0.71 (.81)					-	0.036
7. Age	16.31 (1.22)						-

Note. Significance levels ** *p* < 0.01, *** *p* < 0.001; Care (M): Maternal Care assessed with the Parental Bonding Instrument; Care (P): Paternal care assessed with the Parental Bonding Instrument; Overprotection (M): Maternal overprotection assessed with the Parental Bonding Instrument; Overprotection (P): Paternal overprotection assessed with the Parental Bonding Instrument; SDQ = Strengths and Difficulties Questionnaire; C-P Conflict Index: Parent-Adolescent-Conflict Index.

## Data Availability

Due to ethical concerns, supporting data cannot be made openly available.
